# An Assessment of the Antibacterial Efficacy of the GentleWave System in Comparison to Other Endodontic Irrigation Systems: A Systematic Review

**DOI:** 10.1002/cre2.70268

**Published:** 2026-02-26

**Authors:** Eliza Tolley, Morgan Ziola, Dexter Gross, Meaghan Mannix, Darcy Sayre, João Martins de Mello Neto, Rodrigo R. Amaral

**Affiliations:** ^1^ College of Medicine and Dentistry James Cook University Cairns Queensland Australia; ^2^ Department of Endodontics Nova Southeastern University College of Dental Medicine Fort Lauderdale Florida USA

**Keywords:** antibacterial efficacy, endodontic irrigation, GentleWave system, root canal treatment

## Abstract

**Objectives:**

This systematic review evaluated the antibacterial efficacy of the GentleWave system compared with other endodontic irrigation systems.

**Materials and Methods:**

A comprehensive literature search was conducted across multiple databases (PubMed, MEDLINE, Web of Science, Scopus). The eligibility criteria were formulated using the PICOS framework, focusing on the antibacterial efficacy of the GentleWave system compared to other irrigation methods. A total of 2327 articles were screened, with five meeting the selection criteria for inclusion. Four studies were conducted in vitro, one in ex vivo, and various tooth types were tested. A bias assessment was conducted using the QUIN tool.

**Results:**

Four studies had a low risk of bias, with one study scoring medium risk. All concluded that the GentleWave system significantly reduced microbial counts.

**Conclusions:**

Emerging evidence suggests that GentleWave may provide superior antibacterial efficacy in root canal therapy, making it a compelling option. However, further research is needed to confirm its benefits and promote broader adoption.

## 1 Introduction

Apical periodontitis is an infectious disease caused by microorganisms that colonize the root canal system. To achieve the best possible outcome from endodontic treatment, it is crucial to ideally eliminate or significantly reduce bacterial populations within the root canal to levels that promote healing of the surrounding tissue (Chugal et al. 2017; Siqueira and Rôças 2008). If bacteria persist after chemomechanical preparation, with or without intracanal medication, there is an increased risk of unfavorable treatment outcomes (Chugal et al. 2017; Siqueira and Rôças 2008). Therefore, the primary objective of endodontic treatment is to eliminate bacteria from the root canal system, promoting successful healing and ensuring the long‐term preservation of the tooth (Chugal et al. 2017; Siqueira and Rôças 2008).

Irrigation is vital to successful root canal treatment as it serves multiple crucial functions (Gomes et al. 2023; Haapasalo, Shen, et al. 2014; Boutsioukis and Arias‐Moliz 2022). These include reducing friction, improving the cutting effectiveness of files, dissolving tissue, providing a washing effect, and exhibiting an antimicrobial/antibiofilm effect (Gomes et al. 2023). The main challenge for irrigants is the organization of bacteria in biofilms located in the intricate anatomy of the root canal system, making them challenging to eliminate. Additionally, it is the only way to impact areas of the root canal wall untouched by mechanical instrumentation, especially flat and oval canals (Gomes et al. 2023).

Over the years, various irrigation systems have been developed to enhance the removal of bacteria and debris, aiming to improve the overall success rates of root canal therapy (RCT). Conventional irrigation involves manually delivering solutions into the canal using syringes and needles, which is common due to its simplicity and cost‐effectiveness. However, it is limited in its ability to reach all areas of complex canal anatomy.

In endodontics, sonic and ultrasonic activation systems are used to enhance the cleaning and disinfection of root canals by agitating irrigants. Sonic activation operates at low frequencies (1–6 kHz) using air‐driven or electric handpieces to oscillate a flexible polymer or metal tip, whereas ultrasonic activation utilizes high frequencies (25–30 kHz) generated by piezoelectric or magnetostrictive transducers to vibrate a metal tip (Mozo et al. 2012; Van Der Sluis et al. 2007). Passive ultrasonic irrigation (PUI) is the most used method for activating irrigation solutions. It employs ultrasonic energy to agitate the irrigating solution in the root canal, enhancing debris removal and disrupting bacterial biofilms (Mozo et al. 2012). This method improves solution penetration into complex root canal anatomy, making it popular among endodontists (Mozo et al. 2012). PUI is a variation where a small ultrasonic tip is activated in the canal without significant force, allowing effective cleaning with minimal risk to canal walls (Souza et al. 2019).

The ProUltra PiezoFlow Active Ultrasonic System (Dentsply Sirona) combines ultrasonic activation with continuous flow technology, providing consistent solution flow while delivering ultrasonic energy for potentially superior cleaning (Dentsply 2012). The continuous flow and ultrasonic vibrations aim to enhance debris and bacteria removal from the root canal (Dentsply 2012).

The EndoActivator (Dentsply Sirona) is a cordless, battery‐powered handpiece designed to safely and effectively remove the smear layer and biofilm in root canal systems through cavitation and acoustic streaming, which produces the hydrodynamic phenomenon. It uses non‐cutting plastic tips to enhance root canal debridement after instrumentation without actively engaging the dentin walls, preventing further enlargement of the canals (Advanced Endodontics 2024).

The GentleWave system (Sonendo, Laguna Hills, CA) is a minimally invasive approach to endodontic treatment. It utilizes a combination of procedure fluids and acoustic energy to create a vortex, enhancing the cleaning and disinfection of the root canal system (Haapasalo, Wang, et al. 2014). The GentleWave system is designed to reach complex canal areas, such as lateral canals and dentinal tubules, without requiring mechanical instrumentation like files, reducing the risk of canal wall damage, and enhancing treatment outcomes. The manufacturer suggests minimizing the mechanical preparation size to as small as 20/.06 taper. This innovative approach has been compared to several other endodontic irrigation systems, each with its methodology and mechanism of action (Ordinola‐Zapata et al. 2022; Park et al. 2023; Zhang et al. 2019; Coaguila‐Llerena et al. 2022; Velardi et al. 2022).

Despite increasing interest, the GentleWave system is available only commercially in Canada and the United States. Two recently published reviews compared its antibacterial efficacy with that of other endodontic irrigation systems (Varadan et al. 2025; Lazzarotto et al. 2025). The literature lacks comprehensive systematic reviews and meta‐analyses that compare its antibacterial effectiveness with that of other endodontic irrigation systems. Therefore, this systematic review aimed to evaluate its antibacterial efficacy compared to other currently used endodontic irrigation systems, focusing on bacterial reduction through traditional culture methods, CFU, DNA reads, OTUs, and Next‐Generation Sequencing (NGS). This fills a significant gap in the literature and offers valuable insights that could influence clinical practice and future research in endodontics.

## 2 Materials and Methods

The protocol for this systematic review was developed in accordance with the PRISMA (Preferred Reporting Items for Systematic Reviews and Meta‐Analyses) guidelines (Page et al. 2021). The review was registered with the International Prospective Register of Systematic Reviews (PROSPERO) under CRD42024541775.

### 2.1 Literature Search Strategy

Two independent reviewers, E.T. and M.Z., conducted searches across electronic databases, including PubMed, MEDLINE via Ovid, Web of Science, and Scopus. They targeted articles published between January 2014 and April 2025, applying an English‐only filter. Various Medical Subject Headings (MeSH) terms and keywords were used and combined with the Boolean operators “AND” and “OR” to construct a comprehensive search string, as shown in Table 1. The last search was completed on April 29, 2025.

**Table 1:** Database searches and MeSH terms used.

Database	MeSH terms and search strategy
PubMed	(((“Dental Pulp Cavity” OR “Root Canal Therapy” OR “Dental Pulp” OR “Tooth Apex” OR “Regenerative Endodontics” OR “Endodontics” OR “Tooth Root”[MeSH Terms]) OR (gentlewave OR “gentle wave system” OR “gentle wave” OR “gentlewave system”)) AND (“Edetic Acid” OR Disinfection OR “Antibacterial Agents” OR “Sodium Hypochlorite” OR “Antimicrobial Peptides” OR Bacteria OR “Bacterial Load” OR Biofilms OR “Enterococcus faecalis”[MeSH Terms])) AND (“treatment outcome”[MeSH Terms])
Scopus	(TITLE‐ABS‐KEY (gentlewave OR “Gentle Wave System” OR irrigant* OR irrigat* OR endont*)) AND (TITLE‐ABS‐KEY (“Sodium Hypochlorite” OR naocl OR edta OR “Ethylenediaminetetraacetic acid” OR antimicrob* OR disinfect* OR antibacteria* OR bacteria* OR biofilm)) AND (TITLE‐ABS‐KEY (efficacy OR efficien* OR effect*)) AND PUBYEAR > 2013 AND PUBYEAR < 2025 AND (LIMIT‐TO (SUBJAREA, “DENT”))
Medline Ovid	1. exp Dental Pulp Cavity 2. exp “Root Canal Therapy” 3. exp “Root Canal Preparation” 4. exp Dental Pulp 5. exp Tooth Apex 6. exp Regenerative Endodontics 7. exp Endodontics 8. exp “Tooth Root” 9. 1 or 2 or 3 or 4 or 5 or 6 or 7 or 8 10. exp Edetic Acid 11. exp Disinfection 12. exp Antibacterial Agents 13. exp Sodium Hypochlorite 14. exp Antimicrobial Peptides 15. exp Bacteria 16. exp Bacterial Load 17. exp Biofilms 18. exp Enterococcus faecalis 19. 10 or 11 or 12 or 13 or 14 or 15 or 16 or 17 or 18 20. exp Treatment Outcome 21. gentlewave.mp. 22. gentle wave.mp. 23. 21 or 22 24. 9 or 23 25. 19 and 20 and 24
Web of Science	“Gentle Wave System” OR irrigant* OR irrigant* OR endont* (Keyword Plus ®) AND “Sodium Hypochlorite” OR NaOCl OR EDTA OR “Ethylenediaminetetraacetic acid” OR antimicrob* OR disinfect* OR antibacteria* OR bacteria* OR biofilm (All Fields) AND efficacy OR efficien* OR effect* (All Fields) and 2024 or 2023 or 2022 or 2021 or 2020 or 2019 or 2018 or 2017 or 2016 or 2015 or 2014 (Publication Years) and English (Languages) and Dentistry Oral Surgery Medicine (Web of Science Categories)

**Figure 1:**
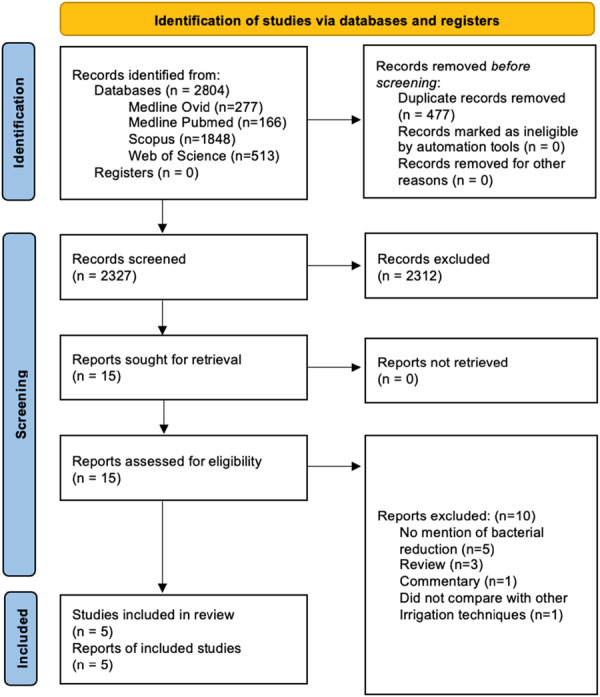
Preferred Reporting Items for Systematic Reviews and Meta‐Analyses (PRISMA) flow‐diagram detailing study selection.

Using a predetermined search strategy, the reviewers identified relevant articles and exported citations into EndNote 21 for reference management. After removing duplicates, three authors (M.M., D.G., and D.S.) independently screened the remaining articles by reviewing their titles and abstracts to determine potential inclusion. Disagreements were resolved through discussion, with a third author (E.T.) consulted if consensus was not achieved. Full‐text evaluations of the selected articles were then conducted. The data collection process is illustrated in the PRISMA flowchart (Figure 1).

### 2.2 Eligibility Criteria and PICO Framework

The inclusion criteria for the articles were formulated using the “PICOS” strategy. The selected studies involved adult human extracted teeth with root canals contaminated with bacteria (P, population) and examined the use of the GentleWave system for canal irrigation (I, intervention). These studies were compared to traditional endodontic irrigation techniques (C, comparison) to evaluate the antibacterial effectiveness of the irrigation systems (O, outcome). The studies included varied designs, encompassing in vitro, in vivo, and ex vivo conditions (S, study design). The primary research question was: How does the GentleWave system compare to other endodontic irrigation systems in reducing intracanal bacterial load during primary RCT?

### 2.3 Inclusion Criteria

The inclusion criteria for this systematic review encompassed studies evaluating root canal treatment using various irrigation systems, including conventional syringe, ultrasonic, sonic, multisonic, and laser activation methods, and comparing their effectiveness in bacterial reduction with the GentleWave system. Eligible study designs included randomized controlled trials, in‐vitro, in‐vivo, and ex‐vivo studies. Additionally, studies were eligible for inclusion if published between January 2014 and April 2024, covering 10 years. This time range was selected to ensure the review reflects current advancements and techniques in endodontics.

### 2.4 Exclusion Criteria

The exclusion criteria included studies not in English and those with inaccessible full text. Furthermore, studies that did not evaluate irrigation systems were excluded. Additionally, case reports, case series, editorials, cohort studies, case‐control studies, and reviews, including systematic reviews, were not considered for inclusion.

### 2.5 Data Extraction

The three authors systematically reviewed each included study, and the following information was extracted and recorded on data collection sheets: authors/year, study design, sample size and tooth type, Gentlewave system protocol, conventional technique protocol, and main results. For articles with missing critical data, attempts were made to contact the authors to obtain the necessary information. To reduce the risk of bias in data extraction, the authors’ names of the included studies were anonymized for the reviewers.

### 2.6 Assessment of Bias Within Studies

Five studies were included in this systematic review. Two reviewers (E.T. and M.Z.) independently assessed the risk of bias in these studies using the Quality Assessment Tool for In Vitro Studies (QUIN tool), as seen in Table 2 (Sheth et al. 2024). Four of the five studies were in vitro, and one was ex vivo. Since a specific tool for assessing ex vivo studies was unavailable, the QUIN tool was also applied to the ex vivo study, given the applicability of many of its criteria. The risk of bias was categorized as “low risk,” “medium risk,” or “high risk” (Sheth et al. 2024).

**Table 2:** QUIN tool method.

1	Clearly stated aims/objectives
2	Detailed explanation of sample size calculation
3	Detailed explanation of the sampling technique
4	Details of the comparison group
5	Detailed explanation of the methodology
6	Operator details
7	Randomization
8	Method of measurement of outcome
9	Outcome assessor details
10	Blinding
11	Statistical analysis
12	Presentation of results

## 3 Results

### 3.1 Study Selection

The combined electronic and hand searches yielded 2327 articles. After screening the titles, abstracts, results, and conclusions, 15 articles were selected for full‐text review based on the eligibility criteria. Of the initial set, 10 articles were deemed ineligible for failing to meet the inclusion criteria. The remaining five articles (Ordinola‐Zapata et al. 2022; Park et al. 2024; Zhang et al. 2019; Coaguila‐Llerena et al. 2022; Velardi et al. 2022) met the eligibility standards (Figure 1).

### 3.2 Characteristics of Included Studies

The five studies analyzed reported that the GentleWave system significantly reduced microbial load and altered microbial colonies more effectively than conventional techniques. Most studies were conducted in vitro, simulating various tooth morphologies. The sample selection in tooth types and morphology varied among the selected studies for review. Two studies focused on mandibular molars, one on maxillary premolars, one on both maxillary and mandibular molars, and one on mandibular incisors (Ordinola‐Zapata et al. 2022; Park et al. 2024; Zhang et al. 2019; Coaguila‐Llerena et al. 2022; Velardi et al. 2022). All studies used permanent teeth without previous endodontic treatment.

The studies used different storage media for collected teeth, including thymol, saline, phosphate‐buffered saline, and sodium azide. All teeth were sterilized, primarily through autoclaving, and instrumented with #10 K‐files to ensure patency was achieved. Notably, Zhang et al. (2019) prepared all their sample teeth with the GentleWave system as part of pre‐treatment before bacteria inoculation. Pre‐operative samples were taken using vortex blue rotary files, followed by sterile paper points (Zhang et al. 2019).

All studies compared the GentleWave system's antibacterial efficacy against conventional irrigation systems. Two studies used PUI as a comparator, while three used a Piezoelectric Active Ultrasonic System (Ordinola‐Zapata et al. 2022; Park et al. 2024; Zhang et al. 2019; Coaguila‐Llerena et al. 2022; Velardi et al. 2022). Outcomes concerning individual tooth morphology types are as follows and are shown in Table 3.

**Table 3:** Summary of results.

Authors, year	Study design	Sample size and tooth type	GentleWave system (GWS) protocol	Conventional technique protocol	Main results
Ordinola‐Zapata et al. (2022)	In‐vitro	22 mandibular incisors.	Glide path K‐files size 10–20 to working length (WL). No rotary instruments were used. First irrigation cycle: 1 min with distilled water. Second irrigation cycle: 4 min NaOCl (3%). Final irrigation with 8% EDTA. Total irrigant = 400 mL/cycle. NaOCl inactivated with 2 mL of 10% sodium thiosulfate for 3 min after disinfection.	The specimens were instrumented until size 35/04, 0.5 mm short of the apex (using the crown‐down technique). Irrigated with 10 mL of 6% NaOCl using a 30 G side vented needle placed at 2–3 mm from WL. Passive ultrasonic irrigation (PUI) with an additional 6 mL of NaOCl was also used to irrigate the canal for 60 s. Final irrigation of 1 mL EDTA (17%).	Both cleaning methods significantly reduced microbial reads post‐cleaning (*p* < 0.0001). PUI was more effective than non‐instrumentation cleaning (*p* = 0.002).
					PUI:
					(pre) = 38,090 ± 4844. (post) = 2813 ± 4410. GWS: (pre) = 36,833 ± 7340. (post) = 14,920 ± 10,873.
Park et al. (2024)	Ex‐vivo	23 mandibular molars.	Pulp chamber and canal orifices were rinsed with 2 mL of 6% NaOCl. Distal canals excluded from instrumentation protocol. Mesial canals enlarged to 20/.04 Vortex Blue file Occlusal platform fabricated with SoundSeal for fluid‐tight seal. Final irrigation protocol: 3% NaOCl for 5 min, distilled water for 30 s, 8% EDTA (neutral pH) for 1.5 min, and final distilled water rinse for 15 s at 45 mL/min flow rate.	Pulp chamber and canal orifices were rinsed with 2 mL of 6% NaOCl. Distal canals excluded from instrumentation protocol. Mesial canals enlarged to 20/.04 Vortex Blue file Ultrasonic Irrigant Activation (UIA): Mesial canals prepared to 30/.04 Vortex Blue file 10 mL of 6% NaOCl using 30 G side‐vented needle 2 mm from WL. #15 ultrasonic file activated at 2 mm from WL using ultrasonic system (power 5/20). Total of 3 mL 6% NaOCl used for 1 min (1 mL irrigation/20 s activation, repeated 3 times). Final irrigation: 4 mL 17% EDTA for 1 min, followed by 3 mL 6% NaOCl.	Bacterial reduction using quantitative real‐time polymerase chain reaction (qPCR):
					UIA: 1.69 ± 0.90 log_10_ reduction. GWS: 2.61 ± 1.16 log_10_ reduction (significantly higher than UIA, *p* = 0.048).
					Operational taxonomic units (OTUs):
					Pre‐treatment: higher OTUs in both groups (UIA: 155 ± 79, GWS: 187 ± 121). Post‐treatment: significant OTU reduction in UIA (*p* < 0.001); minimal change in GWS.
					Specific bacterial reduction:
					UIA: significant reduction in *Eubacterium* (*p* = 0.007). GWS: significant reduction in *Prevotella* (*p* = 0.002).
Zhang et al. (2019)	In‐vitro	20 maxillary and mandibular 1st and 2nd molars.	Hand K‐file #10, #15 to WL, followed by Vortex Blue rotary file #15/.04 to WL. Irrigation: 1 mL of sterile water with a 5 mL syringe and 30 G side‐vented needle after hand and rotary files; needle inserted ≥ 1 mm from WL. Protocol followed: 3 min of 3% NaOCl, 30 s of sterile water, 2 min of 8% EDTA. Final irrigation with sterile water for 15 s. Overall treatment time with GWS per tooth: 5 min and 45 s.	Hand K‐file #10, #15, and #20 to WL. The specimens were instrumented with Vortex Blue file until size 35/04 to WL with 1 mL of sterile water irrigation between each file. Final irrigation protocol used ProUltra PiezoFlow (PF): 3% NaOCl for 1 min per canal, sterile water for 10 s per canal, 8% EDTA for 1 min per canal, sterile water for 10 s per canal. Overall treatment time with the PF system per tooth: teeth with 3 canals ‐ 7 min, teeth with 4 canals ‐ 9 min and 20 s.	Based on qPCR, the average reduction of total bacteria, *streptococci* and *E. faecalis* was 99.67%–99.91% in the GWS group and 97.28%–98.61% in the PF group. These are statistically significant. Range of reduction in individual canals: GWS had consistent reduction (99.27%–99.99%), PF had broader range of reduction (85.77%–99.99%). GWS achieved more predictable and higher microbial DNA removal from root canals.
Coaguila‐Llerena et al. (2022)	In‐vitro	22 permanent mandibular molars.	All teeth were sealed with a barrier (Soundseal, Sonendo). The instrument tip was positioned 1–2 mm above the pulp chamber floor. The irrigation protocol included: − 1‐min cycle with distilled water − 4‐min cycle with 3% NaOCl − 1‐min cycle with 8.5% EDTA − Final 1‐min cycle with distilled water After final irrigation, all canals were flushed with 2 mL of 10% sodium thiosulfate for 3 min to neutralize the residual NaOCl.	Final irrigation, 3% NaOCl was activated in each canal (MB and ML), 2 mm short of the working length, using a 20.02 ultrasonic tip connected to a piezoelectric device (EndoUltra, Vista Dental). A total of 2 mL of NaOCl was activated for 20 s per cycle, repeated three times for a total irrigation time of 1 min. The total volume of NaOCl used per canal was 6 mL. The same procedure was performed with 17% EDTA. After final irrigation, all canals were flushed with 2 mL of 10% sodium thiosulfate for 3 min to neutralize the residual NaOCl.	GWS group mean bacterial reduction:
					Pre‐operative sample: 5.6 molecules/μL (log_10_) Post‐operative sample: 0.44 molecules/μL (log_10_)
					PUI group mean bacterial reduction:
					Pre‐operative sample: 5.95 molecules/μL (log_10_) Post‐operative sample: 1.75 molecules/μL (log_10_) The PUI system demonstrated effective bacterial reduction, though not as pronounced as in the GWS.
Velardi et al. (2022)	In‐vitro	48 first permanent premolars	GWS groups (GWS 1 Minimally Invasive Technique (MIT) and GWS 1 Conventional Instrumentation Technique (CIT)), a #10 hand K‐file to check patency. The GWS protocol involved: − 3% NaOCl for 5 min − Distilled water for 15 s − 17% EDTA for 2 min − Distilled water for another 15 s − After completing the GWS cycle, the canals were treated with 5 mL of 0.5% sterile sodium thiosulfate for 1 min to neutralize the NaOCl. − The canals were then rinsed with sterile saline solution.	PUI groups (PUI 1 MIT and PUI 1 CIT), each tooth was rinsed with 5 mL of 3% NaOCl using a 30‐G side‐vented Maxi‐i‐Probe needle. NaOCl was activated for 1 min in each canal using a size 15 Satelec Sonofile K‐file ultrasonic tip at a power setting of 4 with the ProUltra Piezo Ultrasonic unit. The ultrasonic file was positioned 2 mm short of the root canal length. The canals were irrigated with 5 mL of 3% NaOCl. Next, 5 mL of 17% EDTA was used for irrigation for 1 min, followed by a rinse with 5 mL of 3% NaOCl. This irrigation cycle was repeated twice, totaling 3 min of EDTA action. Afterward, the canals were rinsed with 5 mL of 3% NaOCl and neutralized with 5 mL of 0.5% sterile sodium thiosulfate for 1 min. A final rinse was performed using 5 mL of sterile saline solution.	Sampling time s1:
					*E. faecalis* LTA recovered from all root canals (48/48).
					Sampling times s2 and s3: GentleWave system (GWS): GWS + MIT and GWS + CIT:
					Most effective protocols (no significant difference, *p* > 0.05). *E. faecalis* LTA absent in 42% of root canals.
					Passive ultrasonic irrigation (PUI):
					PUI + CIT more effective than PUI + MIT (*p* < 0.05). Less effective than GWS protocols (*p* < 0.05). *E. faecalis* LTA recovered in 100% of root canals after both PUI protocols.

### 3.3 Descriptive Analysis of the Studies

#### 3.3.1 Irrigation of Mandibular Incisors

Ordinola‐Zapata et al. (2022) studied the biofilm disinfection efficacy of the GentleWave system compared to passive sonic irrigation in mandibular incisors. Using traditional culture methods and NGS, they evaluated bacterial load reduction based on CFUs, DNA reads, and OTUs (Ordinola‐Zapata et al. 2022). The study concluded that passive sonic irrigation significantly reduced microbial load (*p* = 0.002) and induced a more significant shift in microbial community composition (Ordinola‐Zapata et al. 2022).

#### 3.3.2 Irrigation of Mandibular Molars

Within the two studies that focused on mandibular molars, Park et al. (2024) conducted an ex‐vivo study on molars with pulpal necrosis, comparing ultrasonic irrigant activation and GentleWave (Coaguila‐Llerena et al. 2022). The GentleWave system significantly reduced microbial load more than PUI. However, both systems produced similar shifts in microbial diversity. Coaguila‐Llerena et al. (2022) found similar bacterial reduction between GentleWave and PUI in mandibular molar mesial roots.

#### 3.3.3 Irrigation of Maxillary and Mandibular Molars

Zhang et al. (2019) analyzed biofilm removal in maxillary and mandibular molars. Their outcomes demonstrate the GentleWave system significantly reducing total microbial DNA (*p* = 0.007), *Enterococcus faecalis* DNA (*p* = 0.011), and Streptococcus species DNA (*p* = 0.029) (Zhang et al. 2019). The average residual microbial DNA was 0.09% for GentleWave, compared to 1.99% for the ProUltra PiezoFlow system, highlighting superior antimicrobial efficacy (Zhang et al. 2019).

#### 3.3.4 Irrigation of Maxillary First Premolars

Velardi et al. (2022) evaluated the GentleWave system's efficacy on maxillary first premolars, comparing it with PUI. Both the conventional and GentleWave protocols were effective at removing *E. faecalis* lipoteichoic acid (LTA), with similar outcomes between the two approaches (*p* > 0.05) (Velardi et al. 2022). They determined that the GentleWave groups had more canals with undetectable levels of *E. faecalis* LTA after treatment compared to the conventional method testing group (Velardi et al. 2022).

### 3.4 Risk of Bias (Quality Assessment)

The results in Table 4 indicate that four of the five studies were rated as having a “low risk” of bias, while one study was rated as “medium risk” (Sheth et al. 2024).

**Table 4:** Results of risk of bias assessment using the QUIN tool method.

Reference	1	2	3	4	5	6	7	8	9	10	11	12	Score (out of 24)	Risk of bias
Ordinola‐Zapata et al. (2022)	2	1	1	2	2	1	2	2	1	1	2	2	19	Low
Park et al. (2023)	2	2	2	2	2	1	2	2	1	1	2	2	21	Low
Zhang et al. (2019)	2	1	1	1	2	1	1	1	1	1	2	2	16	Med
Coaguila‐Llerena et al. (2022)	2	2	2	1	2	1	2	2	1	1	2	2	20	Low
Velardi et al. (2022)	1	2	2	1	2	1	2	1	1	1	2	2	18	Low

## 4 Discussion

In our systematic review, we included randomized clinical trials (RCTs) and in vitro studies because they can provide high‐quality evidence on the efficacy and biological mechanisms of the interventions under investigation. RCTs provide robust clinical data with minimal bias, while in vitro studies provide mechanistic insights that complement clinical findings.

A meta‐analysis was not performed because the studies presented methodological heterogeneity and lacked sufficient similarity in population characteristics, intervention types, outcome measures, and study design.

The studies reviewed predominantly demonstrate an enhanced antibacterial efficacy related to the GentleWave System compared to conventional irrigation methods. Given the diversity of tooth morphologies analyzed across this review, the GentleWave System's efficacy is proven chiefly superior across multiple clinical scenarios, except for Ordinola‐Zapata et al. (2022) analyzing mandibular incisors. Considering that mandibular incisors are single‐rooted, this exception may propose that using the GentleWave system is indicated in cases of increased tooth morphology complexity. Based on this data, multirooted teeth and teeth with lateral and accessory canals may benefit from the GentleWave System's technology. Namely, reaching anatomy that would typically be inaccessible with conventional irrigation methods, thus achieving superior microbial reduction.

Though the authors did not disclose the reasoning behind the tooth type/s used in each study, the variability within the analysis does exemplify that the GentleWave System can be used across dentition. This consideration becomes relevant where one study lacked specificity in reporting the types of teeth involved in its sample, generalizing the results without providing data for specific tooth subdivisions (Zhang et al. 2019). For instance, Zhang et al. (2019) presented the sample results as a collective that included maxillary and mandibular 1st and 2nd molars without specifying individual outcomes between the two types. Presenting these results can introduce the risk of overgeneralization. Similarly, three articles used broad terms like “mandibular molars” without detailing whether they included first, second, or third molars or a mixture of different molar types. This absence of detail lowered the ability to draw precise conclusions about the effectiveness of irrigation techniques across specific tooth morphologies.

Variations in sample preparation and pre‐instrumentation methods across this review may have also contributed to differing outcomes observed. Ordinola‐Zapata et al. (2022), Park et al. (2024), and Velardi et al.'s (2022) studies utilized standardized preparation protocols across all teeth in their samples, while Zhang et al. (2019) and Coaguila‐Llerena et al. (2022) employed more variable instrumentation techniques between irrigation subgroups, potentially influencing the results seen. It is difficult to accurately compare the irrigation systems’ effectiveness when differing instrumentation protocols have been utilized before their use. Similarly, results across studies may have been influenced by the differing instrumentation protocols used to prepare the same system/s (i.e., the GentleWave System). For instance, differences in how the root canals were initially cleaned and shaped could affect the residual bacterial load, thereby altering the comparative results of the irrigation methods. This variability complicates direct comparisons across studies, as the foundational conditions of the samples are not uniform.

When examining the risk of bias, Zhang et al. (2019) indicated a “medium” level of risk. Although this study demonstrated a highly effective reduction of intracanal bacterial DNA in its GentleWave group, it must be acknowledged that one of the authors who consults Sonendo Inc., which is the manufacturer of GentleWave, has declared a conflict of interest (Zhang et al. 2019). Whilst their study results were still included in this review, data from the other four studies, which indicated no conflict of interest and “low” level of bias risk, were considered and reviewed more thoroughly to prevent any risk of bias.

It is important to note that out of the five studies reviewed, one was conducted ex‐vivo, which is more representative of actual clinical experience (Curtis et al. 2008). Ex‐vivo studies offer a closer approximation to clinical reality than in‐vitro studies, which are more controlled and often less clinically representative (Curtis et al. 2008). The predominance of in‐vitro studies in this review could limit the findings’ applicability to real‐world clinical scenarios, where tissue interactions, biological responses, and clinical skill expertise play a critical role in the success of endodontic treatments.

Within a broader context, the specificity of the research question within the review, which focused exclusively on the antimicrobial efficacy of the GentleWave system, restricted the scope of eligible studies. As a result, studies that explored other benefits of the GentleWave system, such as debris removal or procedural efficiency, were excluded. This narrow focus may have limited the comprehensiveness of the review.

Each of the five selected studies explored the reduction of specific bacterial species, including *Streptococcus*, *Veillonella*, *Campylobacter*, *E. faecalis*, *Parvimonas*, *Prevotella*, *Fusobacterium*, and *Eubacterium* (Ordinola‐Zapata et al. 2022; Park et al. 2024; Zhang et al. 2019; Coaguila‐Llerena et al. 2022; Velardi et al. 2022). Whilst differing from one another in terms of their gram classifications and oxygen requirements, these species share the property of belonging to the nine phyla declared key participants in endodontic infections (Wong et al. 2021). It is understandable that the studies would choose to closely examine changes produced in these integral pathogenic species to not only determine the antibacterial efficacy of the tested irrigation systems but also their capacity to produce successful endodontic outcomes via removing the most integral perpetrators of the infection. However, this limited range of investigated species does not adequately represent the 500–700 types of bacteria present in endodontic infections despite the aforementioned bacteria being the most frequently seen in all endodontic infections (Boutsioukis and Arias‐Moliz 2022). Hence, these available studies may not be representative enough of the spectrum of bacteria that contribute to endodontic infections, and their results are not conducive to determining the antibacterial efficacy of the GentleWave System. A broader selection of bacterial species would have aided the generalizability of the results.

It is important to note that the study by Velardi et al. (2022) solely examined the reduction in *E. faecalis* LTA, not the number of bacteria. LTA is an amphiphile molecule released during gram‐positive bacterial growth and is a potent virulence factor (Wang et al. 2019). According to research gathered by Velardi et al. (2022) to provide contextual background to their study, LTA enables *E. faecalis* adhesion and the establishment of an immune‐mediated inflammatory response to this bacterium. LTA was selected as the study target due to the recent rise in the investigation of LTA in endodontic infections and the lack of experimental evidence to support the use of irrigation systems against this virulence factor (Velardi et al. 2022).

Using LTA as a measure in this context offers advantages and limitations. Whether the success of RCT is based on reducing pathogens to sub‐threshold levels conducive to healing, the evaluation of LTA reduction provides an alternative and clinically relevant, although not definitive, indicator of therapeutic success. A decrease in LTA reflects reduced disease‐causing potential, suggesting that fewer virulence factors are present to perpetuate infection and inflammation (Velardi et al. 2022; Wang et al. 2019). However, LTA reduction may indicate the bactericidal or bacteriostatic effects of irrigation systems and does not directly quantify bacterial load (Wang et al. 2019). Thus, this method shifts the focus from pure antibacterial efficacy to the ability of irrigation systems to mitigate disease progression by targeting virulence factors. This nuanced approach highlights the importance of considering pathogen activity and host interaction, rather than solely pathogen quantity, in assessing therapeutic efficacy. Nonetheless, the inability to directly correlate LTA reduction with bacterial count presents a limitation, underscoring the need for complementary measures to fully understand the mechanisms behind successful endodontic treatment. Whilst examining LTA reduction does hold value in this study's investigation, it perhaps would have served better as an accompaniment to a more exact measurement of *E. faecalis* quantity, such as quantitative real‐time PCR (Bio‐Rad Laboratories Inc. 2024).

As such, the measurement of LTA corresponds to *E. faecalis*’ mitotic state but does not directly relate to the number of bacterial units present at the endodontic infection site. This measurement does not directly represent the quantitative reduction of *E. faecalis* bacteria.

It is also essential to consider that the novelty of the GentleWave system may have contributed to the limited number of available studies, resulting in only five articles being included in the review—just meeting the minimum requirement for a systematic review (Charrois 2015). The keyword “GentleWave” further constrained the pool of eligible studies, potentially excluding relevant research that examined similar irrigation technologies under different terminology.

While this review's findings offer valuable insights into the efficacy of the GentleWave system and other irrigation techniques, limitations must be considered when interpreting the results. These limitations suggest that further research is needed to validate and expand the findings.

The general notion from the included studies reflects that the GentleWave system has greater antibacterial efficacy than conventional irrigation methods and is a compelling basis for further research and potential future changes in clinical practice. This consensus favoring GentleWave for bacterial reduction indicates that this irrigation system may benefit clinical endodontic treatment protocols. Its success in many teeth with various canal morphologies indicates its suitability for generalized use across dentition. The shift to using the GentleWave system in clinical practice could also mean simplifying chemomechanical preparation procedures (as in multiple studies, preparation of the teeth for GentleWave System use was a less rigorous process).

Most of the studies included in this systematic review demonstrated strong methodological rigor, with clearly defined objectives, detailed descriptions of procedures, appropriate randomization strategies, and solid statistical analyses. These factors improve the internal validity and reproducibility of the results. However, several limitations were identified that could affect the overall reliability of the evidence. Specifically, many studies lacked transparency regarding blinding protocols, operator calibration, and the qualifications or consistency of those performing the interventions. Additionally, the limited detail about comparison groups made it challenging to fully assess the validity of the outcome comparisons. These gaps emphasize the need for more standardized reporting practices to enhance the interpretability and generalizability of future research in this area.

The scarcity of experimental evidence in this systematic review necessitates further research for more objective conclusions regarding its antibacterial efficacy and clinical potential. Its clinical application is limited by factors such as tooth morphology variations, sample preparation inconsistencies, and reliance on in vitro studies. It is worth further investigating whether this system can produce higher endodontic treatment success rates. Future studies should aim for more comprehensive reporting and include more clinically representative ex‐vivo and histobacteriological studies to strengthen the GentleWave system's evidence base and minimize confounding factors for the developed outcomes.

## 5 Conclusion

The GentleWave system shows promising clinical potential for improved antibacterial effectiveness in RCT compared to other endodontic irrigation systems. It consistently reduces microbial load across various tooth types. However, further research is needed to confirm its benefits and promote broader adoption.

## Author Contributions

**Eliza Tolley:** data curation, formal analysis, investigation, writing – original draft, software. **Morgan Ziola:** data curation, investigation, writing – original draft, software. **Dexter Gross:** data curation, investigation, writing – original draft, software. **Meaghan Mannix:** data curation, investigation, writing – original draft, software. **Darcy Sayre:** data curation, investigation, writing – original draft, software. **João Martins de Mello Neto:** formal analysis, investigation, methodology, software, validation, visualization, writing – review and editing. **Rodrigo R. Amaral:** formal analysis, investigation, methodology, project administration, resources, supervision, validation, visualization, writing – review and editing.

## Funding

The authors received no specific funding for this work.

## Ethics Statement

This systematic review was conducted using publicly available data from previously published studies. No new data were collected from human participants or animals, and therefore, ethical approval and informed consent were not required. The review adhered to established guidelines for systematic reviews, including transparency, accuracy, and integrity in reporting. All included studies were evaluated for ethical compliance as reported by their original authors.

## Conflicts of Interest

The authors declare no conflicts of interest.

## Data Availability

The data that support the findings of this study are available from the corresponding author upon reasonable request.
